# Glucose Transport through *N*-Acetylgalactosamine Phosphotransferase System in *Escherichia coli* C Strain

**DOI:** 10.4014/jmb.2205.05059

**Published:** 2022-07-04

**Authors:** Hyun Ju Kim, Haeyoung Jeong, Sang Jun Lee

**Affiliations:** 1 Department of Systems Biotechnology and Institute of Microbiomics, Chung-Ang University, Anseong 17546, Republic of Korea; 2 Infectious Disease Research Center, Korea Research Institute of Bioscience and Biotechnology, Daejeon 34141, Republic of Korea

**Keywords:** *N*-Acetylgalactosamine, *agaR*, PTS, adaptive evolution, anaerobic fermentation

## Abstract

When *ptsG*, a glucose-specific phosphotransferase system (PTS) component, is deleted in *Escherichia coli*, growth can be severely poor because of the lack of efficient glucose transport. We discovered a new PTS transport system that could transport glucose through the growth-coupled experimental evolution of *ptsG*-deficient *E. coli* C strain under anaerobic conditions. Genome sequencing revealed mutations in *agaR*, which encodes a repressor of *N*-acetylgalactosamine (Aga) PTS expression in evolved progeny strains. RT-qPCR analysis showed that the expression of Aga PTS gene increased because of the loss-of-function of *agaR*. We confirmed the efficient Aga PTS-mediated glucose uptake by genetic complementation and anaerobic fermentation. We discussed the discovery of new glucose transporter in terms of different genetic backgrounds of *E. coli* strains, and the relationship between the pattern of mixed-acids fermentation and glucose transport rate.

## Introduction

Sugars can be actively transported into microbial cells through various transport systems, including phosphotransferase system (PTS), ATP-binding cassette (ABC) transporter, and cation gradient-driven symporter [1, 2]. PTS, one of the most efficient sugar transport systems, consists of non-sugar-specific common enzymes, namely, enzyme I (EI), phosphohistidine carrier protein (HPr), and sugar-specific enzyme II complexes [3]. In the PTS sugar transport of *Escherichia coli*, EI (encoded by *ptsI*) and HPr (encoded by *ptsH*) are shared; their corresponding sugars are phosphorylated by various enzyme II complexes and transported into the cytoplasm [4, 5].

As the most preferred carbon source, glucose can be introduced into *E. coli* cells through various transporters. For instance, glucose is absorbed by receiving a phosphate group derived from phosphoenolpyruvate (PEP) through glucose-specific PTS. It can be transported and phosphorylated by mannose PTS even though its affinity for glucose is lower than that for glucose PTS [6]. Glucose can also be taken up through maltose PTS and *N*-acetylglucosamine PTS in the absence of IICB^Glc^ [7]. In addition to PTS systems, maltose ABC transporter (Mgl ABC) and galactose permease (GalP) can transport glucose [8-10]; the transported glucose is subsequently phosphorylated by glucokinase in the cytoplasm [3].

Glucose PTS-deficient strains are characterized by severe growth retardation and low glucose uptake [9, 11]. Several studies have been performed to discover an alternative route for transporting glucose by growing a strain that cannot use glucose under various conditions. Crigler *et al*. [2] analyzed the characteristics of *glk*-, *manZ*-, and *ptsG*-deleted *E. coli* cells on a minimal medium containing glucose and reported that glucose can be transported through *N*-acetylglucosamine PTS.

When *E. coli* transports glucose through PTS, a phosphate group derived from PEP is transferred to glucose, and PEP is directly converted into pyruvate. Under anaerobic conditions, mixed acids (*e.g.*, lactic acid, acetic acid, and formic acid) are produced from pyruvate for redox balance [12]. When glucose is transported to non-PTS instead of pyruvate, the PEP pool increases, and succinic acid production increases [11, 13, 14]. We performed short-term adaptive evolution in *ptsG*- and *manX*-deleted *E. coli* strain K-12 under anaerobic conditions and discovered that glucose can be transported by ExuT, a non-PTS pathway; concomitantly, an excess amount of succinate in fermentation products is produced [13].

In this study, growth-coupled experimental adaptation was performed to evolve glucose PTS-deficient *E. coli* C strain, and anaerobically adapted cells were characterized through genome sequencing, genetic complementation, and fermentation. A novel glucose transporter, whose expression is normally repressed by a transcription factor in *E. coli*, was reported, and how the genetic background of microorganisms led to the discovery of the transporter was discussed.

## Materials and Methods

### Bacterial Strains

The *E. coli* strains used in this study are listed in Table 1. *E. coli* C strain was obtained from the Korean Collection for Type Cultures (KCTC) at the Korean Research Institute of Bioscience and Biotechnology (Jeongeup, Korea). P1 *vir* phage was kindly provided by Sankar Adhya at the NIH.

### Chromosome Manipulation

The primers used in this study are listed in Table S1. Mutant *E. coli* strains with a single gene deletion from the Keio collection were obtained from the Open Biosystems (USA). The open reading frames (ORFs) of the targeted genes were replaced by a kanamycin selectable marker [15]. Mutations were transferred to other backgrounds through P1 transduction to prepare isogenic strains. P1 *vir* phage lysates of kanamycin-resistant strain JW1087 (BW25113 Δ*ptsG*) from the Keio collection were used to transduce the C strain to generate HK864 (C strain Δ*ptsG*). If needed, pCP20 was transformed into *E. coli* strain to delete the kanamycin resistance gene from the chromosome by FLP recombinase at 30°C. Subsequently, pCP20 with a temperature-sensitive replication origin was cured at 42°C. P1 *vir* phage lysates of kanamycin-resistant strain JW3100 (BW25113 Δ*agaR*) in the Keio collection were used to transduce HK902 (C strain *ptsG*::FRT) cells to generate the HK912 strain and transfer Δ*agaR* (Aga^-^) mutation. Then, 500 bp upstream and downstream regions of *agaR* and kanamycin-resistant marker gene were fused through overlap PCR to introduce Δ*agaR* (Aga^+^) mutations. Subsequently, PCR products were purified and electroporated into L-arabinose-induced *E. coli* HK902 strain carrying pKD46 for chromosomal integration to generate the HK919 strain.

### Culture Conditions

*E. coli* cells were grown in a LB medium (Cat. No. LB-05, LPS solution, Korea) at 37°C with shaking at 200 rpm. Then, 1 ml of starter culture was inoculated in a 125 ml serum vial that contained 100 ml of fermentation medium and had a butyl rubber stopper [16]. Yeast extract (212750) was purchased from Becton Dickinson (Sparks, USA). D-glucose (G8270), NaHCO_3_ (S6014), NaH_2_PO_4_·H_2_O (S9638), and K_2_HPO_4_ (P3768) were purchased from Sigma-Aldrich (USA). The headspace of the fermentation vials was filled with nitrogen gas, and sodium sulfide (final concentration of 1 mM) was added to quench the dissolved oxygen and obtain strictly anaerobic conditions. An anaerobic fermentation culture was prepared at 37°C with shaking at 200 rpm. When glucose or N-acetylgalactosamine were used as a sole carbon source, cell growth was confirmed in M9 minimal agar (M9 Salts (3 g/l KH_2_PO_4_, 0.5 g/l NaCl, 6.8 g/l Na_2_HPO_4_, and 1 g/l NH_4_Cl), 1 mM MgSO_4_, 0.5 mM CaCl_2_). A colony color in MacConkey agar (Cat. No. 281810, BD Difco, USA) was used to confirm the strains transport glucose or N-acetylgalactosamine. Glucose and N-acetylgalactosamine were added final 0.3%, respectively.

### Analytical Procedure

Cell growth was measured in terms of optical density at 600 nm by using a Libra S70 spectrophotometer (Biochrom, UK). The cell cultures were diluted at 1:10 by using PBS to measure the optical density. Metabolite concentration was determined using an Agilent 1100 series HPLC system (Agilent, USA) with an RI detector (Waters 410 RI monitor, Waters, USA) and Aminex HPX-87H column (300 mm × 7.8 mm, Hercules, Biorad). Sample preparation and analytical methods were performed as previously described [16].

### Genome Analysis

The genomic DNAs of *E. coli* strain were purified using a Wizard Genomic DNA purification kit (Cat. No. A1120, Promega, USA). The genomic sequences of the parental and progeny strains were obtained with an Illumina HiSeq 2500 sequencer. Sequencing data were processed as previously described [13]. Genome sequencing data were deposited in the NCBI BioProject under the accession number PRJNA529314 (SRX5608616, SRX5608617, and SRX5608618). The genomic sequence of *E. coli* C strain (ATCC 8739, NC_010468.1) was used for reference mapping. *agaR* and its regulatory regions were amplified through PCR, and *agaR* mutations were confirmed by Sanger sequencing.

### Transcript Analysis

The transcriptions of *agaV* and *agaB* were analyzed through quantitative real-time PCR (qRT-PCR). *E. coli* C strain, HK864, and HK878 cells were grown anaerobically in the fermentation medium at 37°C. Then, 5 ml of cell culture broth was taken, and the cell pellets were harvested through centrifugation at 3,000 rpm for 10 min. Total RNA was isolated using an RNeasy Mini kit (Qiagen, Germany). qRT-PCR was conducted on a LightCycler 96 (Roche Diagnostics, Germany) by using a RealHelix qPCR kit (QP2-P500, Nanohelix, Korea). Afterward, 5 ng of total RNA was used in qRT-PCR under the following conditions: cDNA synthesis (50°C, 40 min), denaturation (95°C, 12 min), and amplification for 40 cycles (95°C, 20 sec; 60°C, 1 min). Raw fluorescence data were normalized against the expression level of 16S ribosomal RNA and their corresponding expression levels in the wild-type C strain cells.

## Results

### Anaerobic Cell Growth of Glucose PTS-Deficient *E. coli* C Strain

**Fig. 1:**
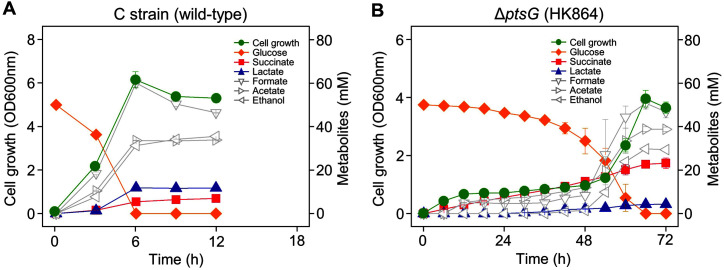
Anaerobic cell growth and fermentation profiles of *Escherichia coli* C strains. (**A**) Wild-type cells and (**B**) Δ*ptsG* cells.

Wild-type *E. coli* C strain cells reached a maximum OD_600nm_ of 6.2 and completely consumed 50 mM of D-glucose in 6 h under anaerobic conditions. The glucose consumption rate of wild-type C strain was 8.3 mM/h, which was measured between 3 and 6 h (Fig. 1A). Mixed acid fermentation produced succinate (5.4 mM), lactate (11.8 mM), formate (60.1 mM), acetate (33.6 mM), and ethanol (33.3 mM; Table 2). Conversely, the C strain Δ*ptsG* cells (HK864 strain) had a long delay in which OD_600nm_ increased to about 0.7 by 18 h, and the change in OD was only about 0.2 until 48 h. The maximum OD_600nm_ at 66 h was 4.0 lower than that of the wild type. About 17 mM D-glucose was consumed gradually until 48 h. After 48 h, glucose was rapidly consumed as cell growth increased. Unlike the wild-type C strain, Δ*ptsG* cells produced a small amount (4.2 mM) of lactate and a large amount (23.2 mM) of succinate at the end of fermentation (Fig. 1B). These results showed that glucose PTS was responsible for the rapid growth and immediate glucose consumption of *E. coli* C strain under anaerobic conditions.

### Accelerated Growth of Δ*ptsG* cells via *agaR* Mutations

**Fig. 2:**
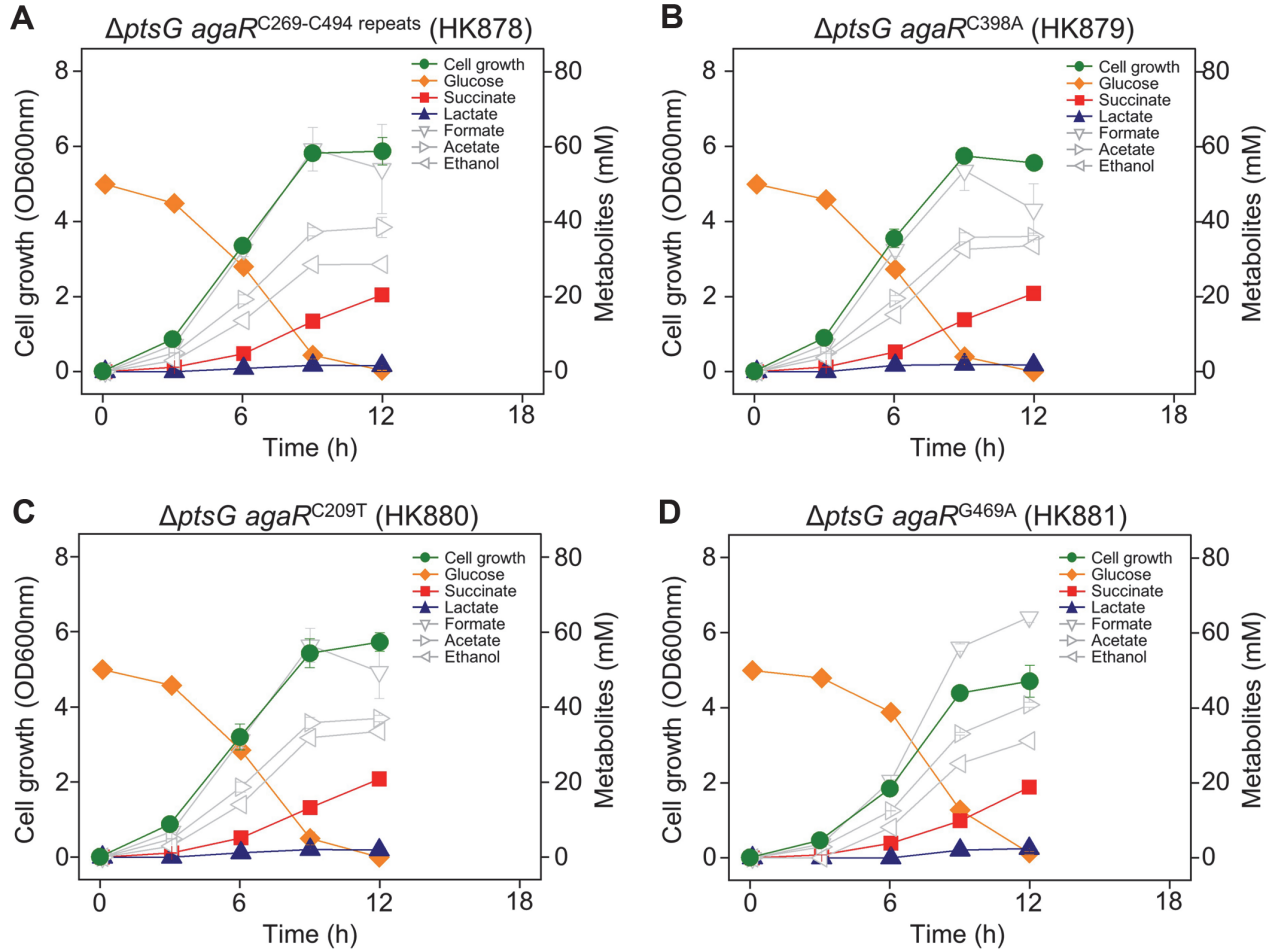
Anaerobic fermentation profiles of adapted progeny cells that evolved from Δ*ptsG* cells.

The culture broth of Δ*ptsG* C strain cells (HK864) grown with a long delay (~72 h) was diluted and spread on the LB medium to obtain progeny cells. Progeny strains (HK878, H879, HK880, and HK881) were grown under the same conditions as the parent cells (HK864) to monitor cell growth and analyze fermentative metabolites. All four progeny strains reached their maximum OD_600nm_ at 9–12 h and completely consumed glucose in 12 h. The glucose consumption rate of the evolved HK878 cells was 4.1 mM/h, which was not as rapid as that of the wild-type C strain (Fig. 2). A low concentration of lactate (1.6–2.5 mM) was produced and the amount of succinic acid (18.9–20.9 mM) increased by 3.5–3.8 times compared with that of the wild-type C strain (Table 2).

The genome sequencing of HK878 and HK881 strains identified mutations in the evolved cells. In the HK878 strain, a repeat of 256 bp (C239–C494) caused premature termination in the ORF of *agaR* and disrupted *agaR*. In the HK881 strain, G469A substitutional mutation in *agaR* caused a missense mutation (Gly157Arg) in the *AgaR* transcriptional factor (Table S2). Sanger sequencing of *agaR* in HK879 and HK880 strains showed C398A and C209T substitutions in the ORF, respectively, causing A133E and A68V missense mutations of *AgaR* proteins.

### Expression Levels of *agaV* in the Evolved Δ*ptsG* Strain

**Fig. 3:**
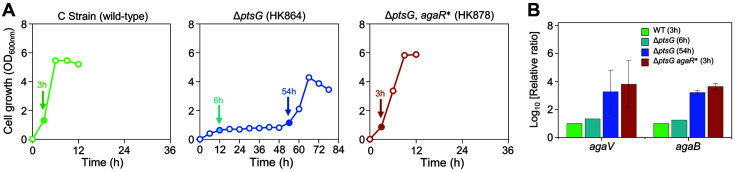
Relative gene expression analysis of *N*-acetylgalactosamine PTS gene in *Escherichia coli* strains. (**A**) Growth curves and sampling points of wild-type C strain, parental Δ*ptsG* cells, and adapted HK878 strain. Arrows indicate sampling points for total RNA isolation for RT-qPCR. (**B**) Relative expression levels of *agaV* and *agaB* in parental Δ*ptsG* cells and adapted HK878 strain.

*agaR* encodes *AgaR*, a transcriptional factor that regulates the expression of a PTS transporting *N*-acetylgalactosamine [17, 18]. As one of *agaR* mutations in adaptive evolution was a premature termination, it was predicted to be a loss-of-function mutation in *agaR*. Therefore, RT-qPCR was performed to confirm whether the gene expression suppressed by the *AgaR* transcriptional regulator increased. Total RNAs were extracted not only from the wild-type strain at 3 h when the glucose consumption rate was maximum but also from the HK864 strain at 6 h with a delayed period and at 54 h when cellular growth was observed. In the HK878 strain, total RNA was extracted at 3 h when the consumption rate of D-glucose was the maximum (Fig. 3A). In the *N*-acetylgalactosamine PTS (Aga PTS), the transcript amount of *agaV*, known as the IIB component, was analyzed. When the *agaV* expression of the HK864 strain was compared with that of the wild type, log_10_ (relative expression ratio) of *agaV* did not change at 6 h, but it significantly increased by about 3.3-fold at 54 h. In the HK878 strain, log_10_ (relative expression ratio) of *agaV* increased 3.8 times compared with that of the wild type (Fig. 3B). These results indicated that the expression of the Aga PTS transporter gene in the Δ*ptsG* strain increased during experimental evolution; furthermore, the transcription of Aga PTS genes increased in the evolved HK878 strain.

### Glucose Transport through *N*-acetylgalactosamine PTS

**Fig. 4:**
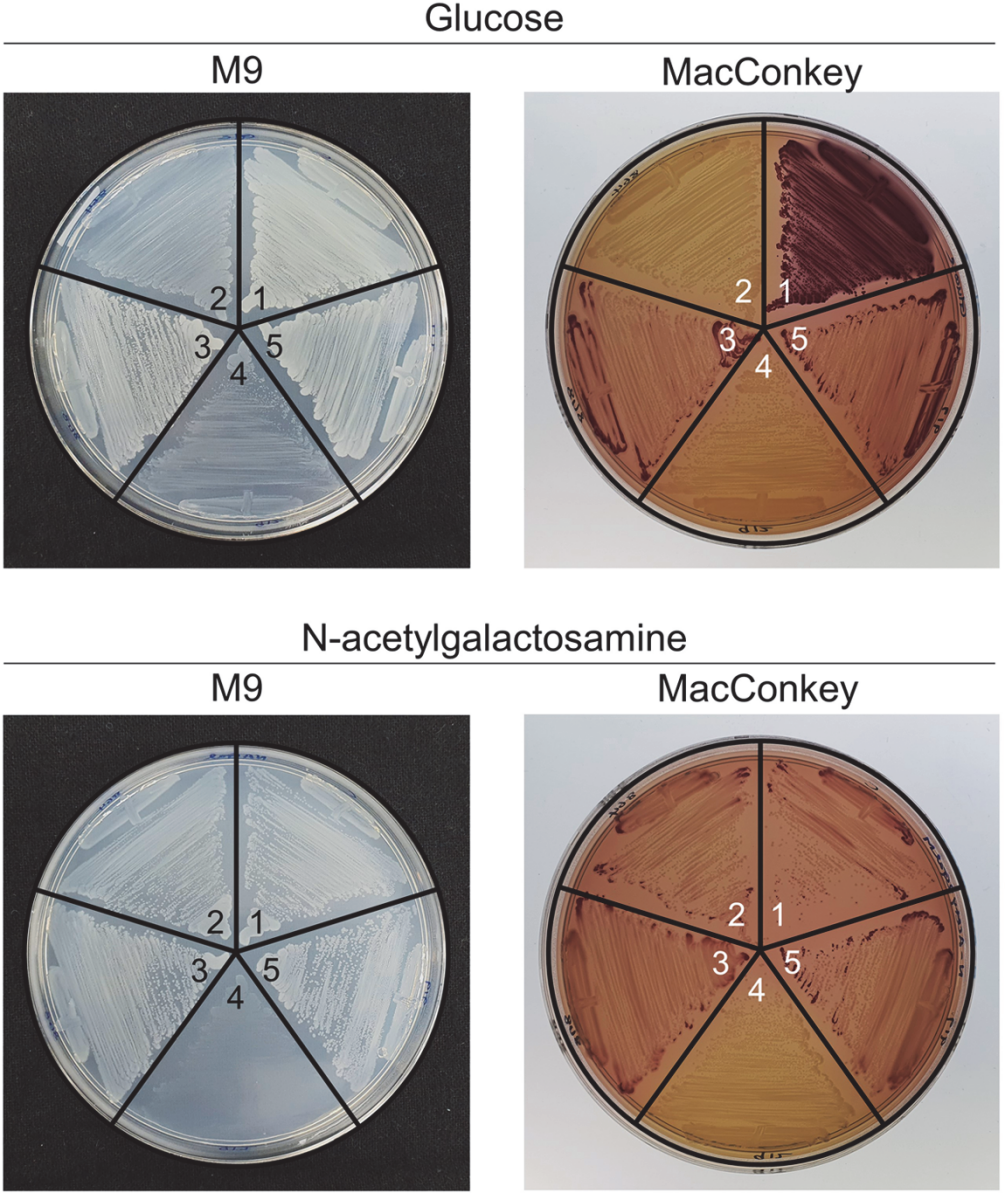
Growth of various *agaR* mutant cells derived from Δ*ptsG* cells. Cells were streaked on M9 minimal agar and MacConkey agar containing D-glucose or *N*-acetylgalactosamine. 1, C strain (wild type); 2, HK864 (Δ*ptsG*); 3, HK878 (Δ*ptsG*, agaR*); 4, HK912 (Δ*ptsG*, Δ*agaR* [Aga^-^]); 5, HK919 (Δ*ptsG*, Δ*agaR* [Aga^+^]).

We genetically verified whether the loss-of-function mutation in *agaR* could transport glucose into cells and whether the Aga PTS structural genes were involved in glucose transport. We checked the uptake and metabolism of glucose and *N*-acetylgalactosamine by using M9 minimal and MacConkey media containing the corresponding sugars (Fig. 4).

In the glucose-containing medium (M9 minimal agar), wild-type C strain could grow fully. However, Δ*ptsG* cells (HK864) grew poorly because of the absence of the major glucose transporter. The growth of Δ*ptsG* *agaR* mutant cells (HK878), which evolved from Δ*ptsG* cells (HK864), was restored. While Δ*ptsG* Δ*agaR* (Aga^-^) cells (HK912) poorly grew in M9 minimal glucose agar, the growth of Δ*ptsG* Δ*agaR* (Aga^+^) cells (HK919) was restored. In glucose-containing MacConkey agar, strong red wild-type colonies showed efficient glucose uptake because of the presence of PtsG, an efficient glucose transporter. White colonies of Δ*ptsG* (HK864) and Δ*ptsG* Δ*agaR* (Aga^-^; HK912) exhibited poor glucose uptake. Slightly red colonies of Δ*ptsG* *agaR* mutant cells (HK878) and Δ*ptsG* Δ*agaR* (Aga^+^) cells showed significant glucose uptake.

The same set of cells was streaked on M9 and MacConkey agar containing *N*-acetylgalactosamine. Only Δ*ptsG* Δ*agaR* (Aga^-^) cells (HK912) could not grow on M9 minimal agar containing *N*-acetylgalactosamine. Red colonies on MacConkey agar containing *N*-acetylgalactosamine showed *N*-acetylgalactosamine uptake and utilization in wild-type C strain, Δ*ptsG* strain (HK864), Δ*ptsG* *agaR* mutant strain (HK878), and Δ*ptsG* Δ*agaR* (Aga^+^) strain (HK919). These data revealed that Δ*ptsG* cells could efficiently have glucose uptake through *agaR* null mutations via Aga PTS compared with those via other minor glucose transporters.

## Discussion

Under anaerobic conditions, *E. coli* C strain cannot uptake glucose and grow very slowly when glucose-specific PTS is disrupted. Through experimental evolution, glucose-PTS-deficient cells could consume glucose and restore growth through *agaR* mutations. Genetic approaches confirmed that glucose was transported by Aga PTS activation.

**Fig. 5:**
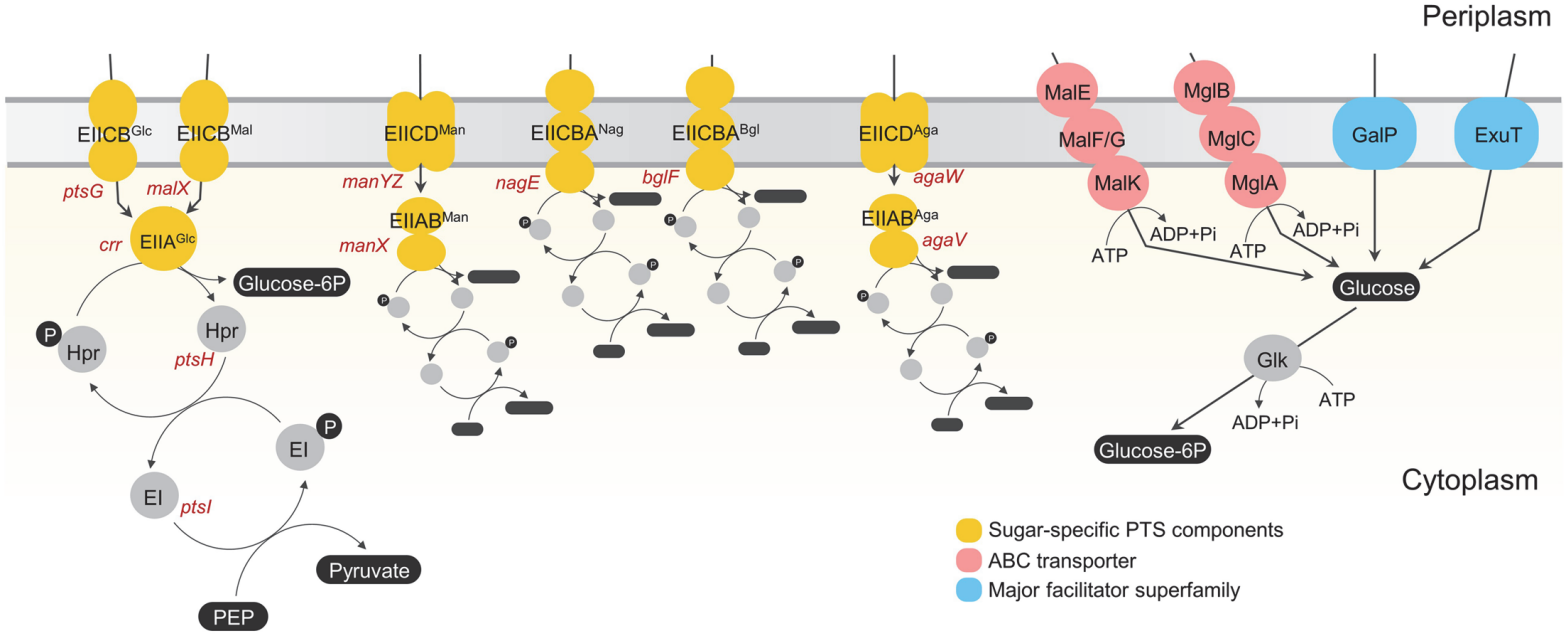
D-Glucose transporters in *Escherichia coli*. Five different sugar PTSs, including Aga PTS reported in this study, could perform D-glucose uptake. Two ABC transporters and two facilitators are reported.

The glucose transport system in *E. coli* is known as follows. There are glucose PTS [19], mannose PTS [20], maltose PTS [21], and *N*-acetylglucosamine PTS [2]. Non-PTS transporters include Mal ABC transporter [3], ExuT [13], Mgl ABC transporter [22], and GalP [9, 23] (Fig. 5). The Aga PTS identified in this study was added. The comparison of the operon structures and gene sequences of the known glucose transporters in the genomes of K-12 strain (MG1655) and C strain (ATCC 8739) confirmed that some genes differed (Fig. S1). In C strain, a loss-of-function mutation was identified because of the IS1 insertion into *manX* among mannose PTS genes. In addition, *malX*, one of the maltose PTS genes, was found to cause the premature termination of the gene product because of nucleotide deletion.

In case of the K-12 strain, the 2.3 kb region, including *agaWEF*, a part of the *N*-acetylgalactosamine PTS gene, is deleted. *N*-acetylgalactosamine cannot be utilized because of the deletion of 2.3 kb, including the *agaF* region encoding EIIA in the K-12 strain [18, 24, 25]. Although *E. coli* K-12 strain has been studied as a standard strain for a long time, glucose transport via Aga PTS may not be observed in the K-12 strain because of this genetic defect.

When glucose is transported through PTS, equimolar PEP is converted to pyruvate. Under anaerobic conditions, *E. coli* performs mixed acid fermentation to produce succinic acid, lactic acid, acetic acid, and formic acid. When glucose is transported through non-PTS, succinic acid production increases because of the increase in the PEP pool [13]. However, when glucose was transported through Aga PTS in the evolved C strains, succinic acid production increased to about 3.5–3.8 times that of the wild type, and lactic acid production decreased (Table 2). The glucose consumption rate of wild-type C strain was 8.3 mM/h (Fig. 1A), but the glucose consumption rate of the evolved strain (HK878) transported by Aga PTS was reduced to 4.1 mM/h (Fig. 2A). The production pattern of metabolites is determined by the intracellular PEP/pyruvate pool ratio, which is changed by the glucose transport rate. Therefore, succinic acid production in the evolved cells increased because of the slower transport rate of glucose by Aga PTS than that by the efficient glucose-specific PTS.

When the K-12 strain was unable to perform glucose uptake due to the deletion of *ptsG*, the loss-of-function mutations of transcriptional regulators such as Mlc and ExuR are frequently obtained through laboratory evolution [13]. The expression of other sugar transporter genes is increased by the inactivation of transcriptional regulators; thus, glucose can be transported through some transporters with a low sugar specificity. Glucose can also be transported into cells through various transporters with different sugar affinities and specificities (Fig. 5). In this study, we discovered a novel glucose transporter known as *N*-acetylgalactosamine transporter that was hidden by transcriptional regulators through experimental evolution in *E. coli*. Microorganisms have metabolic and biochemical potential in their genomes to grow in various environments. Microbial cells can adapt rapidly to their environment through mutations in regulatory networks of metabolism.

## Supplemental Materials

Supplementary data for this paper are available on-line only at http://jmb.or.kr.

## Figures and Tables

**Table 1:** Bacterial strains, phages, and plasmids used in this study.

Name	Relevant genotypes or characteristics	Reference or source
*E. coli* K-12		
JW1087	BW25113 Δ*ptsG*::FRT-KmR-FRT	Keio collection
JW3100	BW25113 Δ*agaR*::FRT-KmR-FRT	Keio collection
*E. coli* C		
ATCC 8739	Wild-type C strain obtained from	KCTC KCTC 2571
HK864*	ATCC 8739 Δ*ptsG*::FRT-KmR-FRT	This study
HK878*	HK864 *agaR* (C239-C494 tandem repeats)	This study
HK879	HK864 *agaR* (C398A substitution)	This study
HK880	HK864 *agaR* (C209T substitution)	This study
HK881*	HK864 *agaR* (G469A substitution)	This study
HK902	ATCC 8739 Δ*ptsG*::FRT	This study
HK912	HK902 Δ*agaR*::FRT-KmR-FRT, Δ*agaWEF*A (Aga−)	This study
HK919	HK902 Δ*agaR*::FRT-KmR-FRT (Aga^+^)	This study
Phage		
P1 *vir*	*vir* mutations	S. Adhya
Plasmid		
pCP20	Temperature-sensitive plasmid with an FLP recombinase capable of recognizing the FRT sequence, ApR	[26]
pKD46	Temperature-sensitive plasmid expressing a lambda RED recombinases, ApR	[27]

* Whole genomic sequencing was performed.

**Table 2:** Fermentation profiles of *E. coli* C strain-derived *agaR* mutant cells.

Strain	Genotype	Fermentation time (h)^a^	OD_600nm_	Concentration (mM)					

				D-glucose^b^	Acetate	Ethanol	Formate	Lactate	Succinate
ATCC 8739	Wild-type C strain	6	5.4 ± 0.1	ND^c^	32.4 ± 0.4	29.2 ± 0.2	54.2 ± 0.9	17.4 ± 0.1	6.2 ± 0.0
HK864	Δ*ptsG*	66	4.0 ± 0.2	ND	38.9 ± 1.1	29.8 ± 2.2	50.8 ± 2.7	4.2 ± 0.5	22.7 ± 1.6
HK878	Δ*ptsG* *agaR* (239-494 tandem repeats)	12	5.9 ± 0.4	0.4 ± 0.0	38.5 ± 2.7	28.6 ± 3.5	54.1 ± 11.9	1.6 ± 0.2	20.5 ± 0.5
HK879	Δ*ptsG* *agaR* (C398A)	12	5.6 ± 0.1	ND	36.1 ± 0.9	33.7 ± 0.5	43.3 ± 6.9	1.8 ± 0.2	20.9 ± 0.6
HK880	Δ*ptsG* *agaR* (C209T)	12	5.7 ± 0.2	ND	37.0 ± 0.9	33.5 ± 1.4	49.4 ± 7.1	1.9 ± 0.6	20.9 ± 0.7
HK881	Δ*ptsG* *agaR* (G469A)	12	4.7 ± 0.4	1.3 ± 0.3	40.9 ± 0.6	31.2 ± 0.6	64.2 ± 1.4	2.5 ± 0.2	18.9 ± 0.2
HK919	Δ*ptsG* Δ*agaR* (Aga^+^)	9	5.8 ± 0.1	ND	39.8 ± 0.1	36.2 ± 0.1	55.0 ± 1.3	2.2 ± 0.0	16.8 ± 0.2
HK912	Δ*ptsG* Δ*agaR* (Aga−)	108	2.2 ± 0.1	ND	31.5 ± 0.6	11.2 ± 0.6	14.4 ± 0.8	3.1 ± 0.5	57.2 ± 1.0

^a^Fermentation time (h) when glucose was completely consumed or less than 2.5 mM.

^b^Residual D-glucose concentration; 50 mM glucose was added initially in the medium.

^c^ND, not detected
