# Supplementary material for: Glucose Transport through N-Acetylgalactosamine Phosphotransferase System in Escherichia coli C Strain
Source: J Microbiol Biotechnol. 2022 Jul 4;32(8):1047–53. doi: 10.4014/jmb.2205.05059 (PMC9628945; doi:10.4014/jmb.2205.05059)
Supplement: Supplementary file 1 [file jmb-32-8-1047-supple.pdf]

Supplementary Data

Glucose Transport through *N*-Acetylgalactosamine Phosphotransferase System in *Escherichia coli* C Strain

Hyun Ju Kim[1], Haeyoung Jeong[2], and Sang Jun Lee[1]*

[1]Department of Systems Biotechnology and Institute of Microbiomics, Chung-Ang University, Anseong 17546, Republic of Korea

[2]Infectious Disease Research Center, Korea Research Institute of Bioscience and Biotechnology, Daejeon 34141, Republic of Korea

*Corresponding author : sangjlee@cau.ac.kr

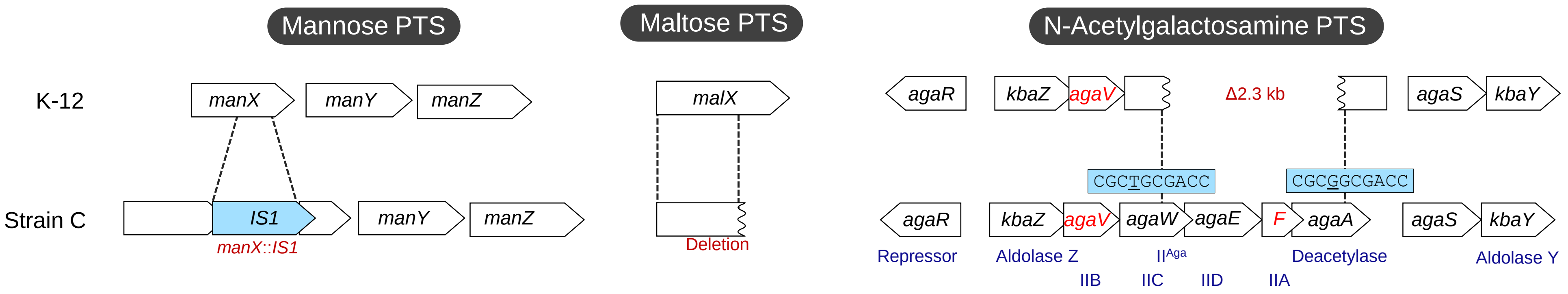

Fig. S1. Comparison of *manXYZ* operon, *malX*, and *N*-acetylgalactosamine PTS gene cluster between *Escherichia coli* C strain (ATCC 8739) and *E. coli* K-12 (MG1655).

Table S1. Primers used in this study.

Name	Sequence (5'→3')
ptsG-F	CAACTGCGCGATGCTGCGCGTTATGTCC
ptsG-R	GTCGCTCAGTCCACCGGTACACCTATCC
agaR-F	GAAACATAATGAAAGTCACTGAAACGAG
agaR-R	GGGATGGATGAACGATGAAAACACGCTG
agaV-RTF	CCGAAGATCCGGTACAACAA
agaV-RTR	TTTTGCAGCGTCCAGAAAC
agaB-RTF	TGATAACCGTCTGGTTCATGG
agaB-RTR	CGACTACCAGCAGATTTGCAC
16S-RTF	CAGCAGCCGCGGTAATAC
16S-RTR	ACCAGGGTATCTAATCCTGT
Kdel-F	TACGCTGGAACTGGTGTGGATGGGGCTG
Kdel-R	CATCGCGTCGGTGATCAGCACGATTCTC
manX-100up	GATATCTAAAATAAATCGCGAAACGCAG
manX-100dn	GATAAATACCAGCACAATTTGAAGAGTG
KmR-ATGout	ACCTGCGTGCAATCCATCTTGTTCAATCAT

Table S2. Genome analysis of parental *E. coli* C strain HK864 and evolved progeny HK878 and HK881 strains.

Strain	Genotype	Reads	Bases	Reads (trimmed)	Bases (trimmed)	Avg. length (trimmed)	Reads mapped	% Reads mapped	Fraction of reference covered	Avg. coverage
HK864	Δ*ptsG*	21,1167,368	3,188,574,286	12,432,204	1,708,391,660	137.4	12,342,960	99.3	1.0	357.2
HK878	Δ*ptsG agaR* (239-494 tandem repeats)	22,559,820	3,406,532,820	13,694,278	1,890,262,836	138.0	13,577,186	99.1	1.0	394.7
HK881	Δ*ptsG agaR* (G469A)	20,136,926	3,040,675,826	12,083,798	1,667,284,262	138.0	11,998,027	99.3	1.0	348.7
